# Refractory myelodysplasia cutis in a patient with progression to acute myeloid leukaemia^☆^

**DOI:** 10.1016/j.abd.2025.501185

**Published:** 2025-08-13

**Authors:** Miguel Mansilla-Polo, Daniel Martín-Torregrosa

**Affiliations:** a Department of Dermatology, Hospital Universitario y Politécnico La Fe, Comunidad Valenciana, Valencia, Spain; b Department of Dermatology, Instituto de Investigación Sanitaria (IIS) La Fe, Comunidad Valenciana, Valencia, Spain

*Dear Editor,*

In 1964, Dr Robert D Sweet reported eight patients with acute febrile neutrophilic dermatosis, later named Sweet Syndrome (SS). Histopathologically, the lesions were characterized by a dermal inflammatory infiltrate rich in neutrophils.^1^ Subsequently, it was observed that up to 20% of patients with SS were associated with malignancy, especially hematological malignancies and specifically of myeloid lineage (acute myeloid leukaemia and Myelodysplastic Dyndrome [MDS]).^2^ In 2005, Requena et al. first reported that some cases of SS did not have neutrophils with normal reactive characteristics but were immature. These mononuclear cells looked more like histiocytes and immunohistochemically expressed Myeloperoxidase (MPO), CD33, CD163, CD68, an immunophenotype consistent with myeloid precursors, and myelomonocytic cells. This histological variant of SS was termed Histiocytoid Sweet Syndrome (HSS) and was subsequently shown to be more common in SS associated with hematological processes than in idiopathic or reactive SS.^3^ More recent findings in the last decade have attempted to further investigate neutrophilic dermatoses associated with MDS. Based on the studies of the group of Vignon-Pennamen et al., the term Myelodysplasia Cutis (MC) has been proposed to refer to myeloid dermatoses in the spectrum between SS and leukemia cutis in patients with MDS. Specifically, the precise definition of MC would represent patients with MDS, lesions compatible with SS, with a dermal infiltrate composed of immature, non-blastogenic myeloid cells, and their clonality is exactly the same as that associated with myelodysplastic cells in the bone marrow. Thus, MC in the skin would be the expression of a clonal process in the bone marrow, with identical mutations to the bone marrow.^4^ This concept would explain why MC is generally corticosteroid dependent, with usually increasing doses (unlike common SS, which generally responds well to corticosteroids). In fact, the most effective treatments reported for MC are those commonly used for MDS, such as demethylating agents (especially azacitidine and decitabine) and, ultimately and as a curative treatment, allogeneic Hematopoietic Stem Cell Transplantation (allo-HSCT).^4^, ^5^

We present a 68-year-old male patient with a history of high-risk MDS (mutations in DMT3A, EZH2 and TP53) who was recently diagnosed and treated of MC in our dermatology department. This patient initially presented (Fig. 1) with lesions compatible (and histologically confirmed) with panniculitis, specifically neutrophilic lobular panniculitis. After an initial response to prednisone, when the dose was reduced to <10 mg/day, the patient continued to develop new nodules and ulcers resembling pyoderma gangrenosum, especially in areas of trauma (canals, biopsies, etc.) (Fig. 2), demonstrating a prominent pathergy phenomenon. In view of these lesions, a diagnosis of MC was proposed and treatment with weekly subcutaneous azacitidine 75 mg/m^2^ was started in collaboration with the hematology department. Histopathological findings (Fig. 3) revealed that the dermal infiltrate was rich in cells of histiocytic habitus, and immunohistochemistry showed that the cells combined myeloid and monocytic immunophenotypes, with expression of both MPO and CD163 and negativity for CD3, PAX5, CD79a, CD34, CD43, CD56 or CD117. No histopathological or immunohistochemical findings compatible with leukemia cutis were found in the samples examined, which was also inconsistent with the response to corticosteroids. Genetic testing of skin and bone marrow for the UBA1 gene was requested and the diagnosis of VEXAS (Vacuoles, E1 enzyme, X-linked, Autoinflammatory, Somatic) syndrome was excluded. These findings and the patient's evolution confirmed the diagnosis of MC. Despite an initial response to azacitidine, the patient continued to develop new lesions. In an attempt to reduce these neutrophilic lesions, treatment with cyclosporine and anakinra was started successively on a compassionate basis, both without response. Due to the patient's age and comorbidities, allo-HSCT was declined. Three months after diagnosis, the patient progressed systemically to acute myeloid leukemia and died of the disease despite multiple cycles of chemotherapy.

**Fig. 1:**
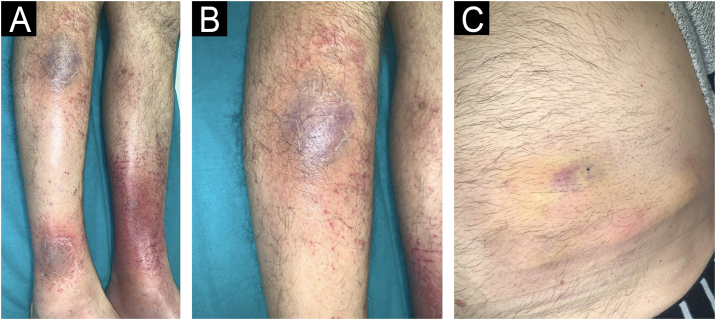
Clinical appearance of the initial skin lesions. Subcutaneous nodules on both lower limbs and left hemiabdomen (in the area of heparin administration, demonstrating a phenomenon of pathergy). Histopathology of the leg lesions showed neutrophilic lobular panniculitis.

**Fig. 2:**
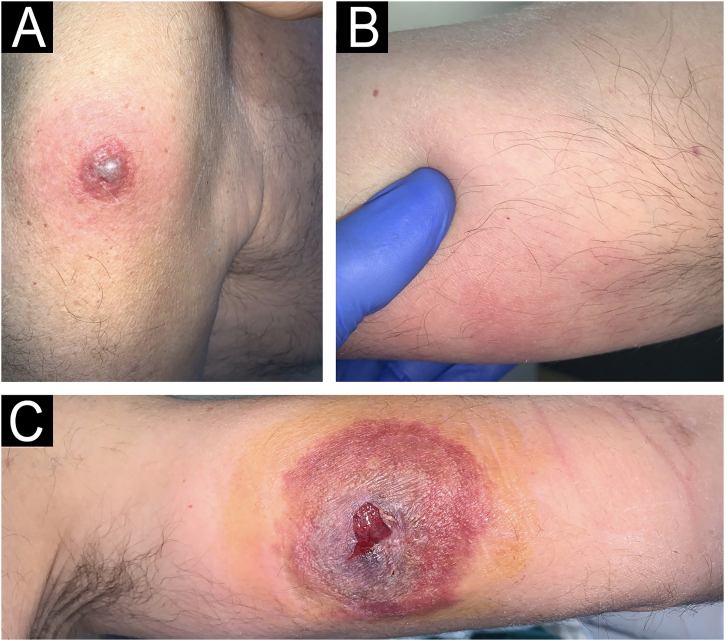
New skin lesions secondary to pathergy. (A) Erythematous violaceous plaque on the left shoulder around vaccine application. (B) Subcutaneous nodule on the medial aspect of the right arm in the area of support. (C) Ulcer resembling pyoderma gangrenosum on the inner side of the left arm in the peripheral route. Histopathology of the left shoulder lesion showed histiocytoid sweet syndrome.

**Fig. 3:**
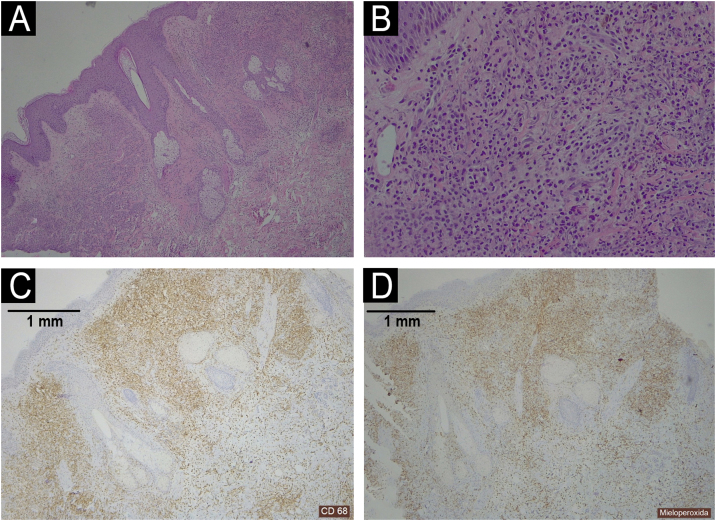
Histopathology of a shoulder lesion (panel A Haematoxylin-Eosin 40×; panel B Haematoxylin-Eosin 200×; panel C CD68 40×; panel D MPO 40×). A dermal infiltrate was rich in atypical cells of histiocytic habitus, arranged in clusters and infiltrating collagen bundles (panel A‒B). Immunohistochemistry showed that the cells combined myeloid and monocytic immunophenotypes, with expression of both CD68 (panel C) and MPO (panel D). Lymphoid markers (CD3, CD79a, CD63 and PAX5) were negative. CD34, CD56, CD117 and CD138 were also negative.

The purpose of this article is to draw the attention of the scientific community to the existence of MC. Particularly striking in MC is the marked phenomenon of pathergy (hence the need to avoid invasive procedures), the frequent corticodependence (its treatment is that of MDS and not, as usual, that of neutrophilic dermatoses), and the need to rule out the presence of VEXAS syndrome, given its known and reported association. This is a recently described autoinflammatory disorder caused by somatic mutations in the UBA1 gene. It is characterized by systemic inflammation, skin lesions (characteristically erythematous oedematous plaques with the appearance of neutrophilic dermatoses), and hematological abnormalities. VEXAS is strongly associated with MDS, with many patients presenting with cytopenia’s and dysplastic bone marrow changes, linking the inflammatory and hematological components through defective ubiquitination and protein homeostasis.^4^, ^5^

## Authors' contributions

Miguel Mansilla-Polo: Managed clinical treatment and procedures, and wrote the initial version of the article.

Daniel Martín-Torregrosa: Supervised the work.

## Research data availability

Does not apply.

## Scientific Associate Editor

Hiram Larangeira de Almeida Jr.

## Financial support

No specific funding was received from any bodies in the public, commercial, or not-for-profit sectors to carry out the work described in this article.

## Conflicts of interest

None declared.

## References

[1] Sweet R.D. (1964). An acute febrile neutrophilic dermatosis. Br J Dermatol.

[2] Zheng S., Li S., Tang S., Pan Y., Ding Y., Qiao J. (2020). Insights into the characteristics of sweet syndrome in patients with and without hematologic malignancy. Front Med (Lausanne).

[3] Requena L., Kutzner H., Palmedo G., Pascual M., Fernández-Herrera J., Fraga J. (2005). Histiocytoid sweet syndrome: a dermal infiltration of immature neutrophilic granulocytes. Arch Dermatol.

[4] Vignon-Pennamen M.D., Battistella M. (2024). From histiocytoid sweet syndrome to Myelodysplasia Cutis: history and perspectives. Dermatol Clin.

[5] Whittington C.P., Ross C.W., Ramirez J.A., Lowe L., Brown N., Hristov A.C. (2024). Myelodysplasia Cutis. Arch Pathol Lab Med.

